# Abdominal-only Compression Garments Reduce Orthostatic Tachycardia and Improve Symptoms in Patients With Postural Orthostatic Tachycardia Syndrome

**DOI:** 10.1016/j.cjca.2025.11.038

**Published:** 2025-12-01

**Authors:** Kate M. Bourne, Kavithra Karalasingham, Tanya Siddiqui, Bianca Mammarella, Aishani Patel, Derek V. Exner, Robert S. Sheldon, Satish R. Raj

**Affiliations:** a Department of Cardiac Sciences, Libin Cardiovascular Institute, Cumming School of Medicine, University of Calgary, Calgary, Alberta, Canada; b Vanderbilt Autonomic Dysfunction Center, Division of Clinical Pharmacology, Department of Medicine, Vanderbilt University Medical Center, Nashville, Tennessee, USA

**Keywords:** abdominal compression, heart rate, nonpharmacologic treatment, postural orthostatic tachycardia syndrome, symptoms

## Abstract

**Background:**

Compression garments are a relatively inexpensive and easy-to-implement treatment, but the longer term benefits of abdominal compression in a real-world setting are not known. In this study we sought to evaluate commercially available abdominal compression garments in a real-world setting in patients with postural orthostatic tachycardia syndrome (POTS).

**Methods:**

Participants completed four 10-minute active stand tests, with and without an abdominal compression garment, in the morning (Test #1: AM-OFF; Test #2: AM-ON) and afternoon (Test #3: PM-ON; Test #4: PM-OFF). Participants held medication that could augment heart rate (HR) and blood pressure during this 1-day study. A Holter monitor was used to record HR and participants recorded their symptoms using the Vanderbilt Orthostatic Symptom Score at the end of each standing test (range 0–90, where 0 = no symptoms). Continuous data are presented as median (25th-75th percentile).

**Results:**

Standing HR (103 [91–114] vs 118 [99–134], *P* = 0.002) and change in HR with upright posture compared with supine (27 [19–42] vs 41 [26–48], *P* < 0.001) were reduced, and symptoms improved (29 [16–45] vs 35 [25–60], *P* = 0.005), during AM-ON compared with AM-OFF. When the garment was removed after several hours of use (PM-ON vs PM-OFF), standing HR (*P* = 0.04), ΔHR (*P* = 0.01), and symptoms (*P* = 0.02) increased.

**Conclusions:**

Commercially available abdominal compression garments reduced HR and improved symptoms both acutely and after several hours of use. Abdominal compression garments may provide a good alternative to full waist-high compression garments in patients with POTS.

**Clinical Trial Registration:**

NCT04881318.

## RÉSUMÉ

**Contexte:**

Les vêtements de compression constituent un traitement relativement peu coûteux et facile à mettre en œuvre, mais les bienfaits à long terme de la compression abdominale en contexte réel ne sont pas connus. Nous avons cherché à évaluer en contexte réel des vêtements de compression abdominale disponibles sur le marché chez des patients atteints d’un syndrome de tachycardie orthostatique posturale (STOP).

**Méthodologie:**

Les participants devaient réaliser quatre tests de 10 minutes de passage en position debout, avec ou sans vêtement de compression abdominale, soit le matin (test n° 1 : AM-OFF, test n° 2 : AM-ON) et l’après-midi (test n° 3 : PM-ON, test n° 4 : PM-OFF). Durant cette étude d’une journée, les participants ont cessé de prendre les médicaments pouvant augmenter la fréquence cardiaque et la pression artérielle. Un moniteur Holter a été utilisé pour enregistrer la fréquence cardiaque et les participants notaient leurs symptômes à l’aide du score VOSS (*Vanderbilt Orthostatic Symptom Score*) à la fin de chaque test de passage en position debout (plage de 0 à 90; 0 = absence de symptôme). Les données continues sont présentées sous forme de médianes (25^e^, 75^e^ percentiles).

**Résultats:**

Comparativement à la position couchée, on a observé une baisse de la fréquence cardiaque en position debout (103 [91 à 114] *vs* 118 [99 à 134], *p* = 0,002) et de la variation de la fréquence cardiaque en position debout, (27 [19 à 42] *vs* 41 [26 à 48], *p* < 0,001); par ailleurs, les symptômes se sont atténués (29 [16 att so*vs* 35 [25 à 60], *p* = 0,005) pendant le test AM-ON, comparativement au test AM-OFF. Lorsque le vêtement a été retiré aprrenplusieurs heures ddures ddsieu (PM-ON *vs* PM-OFF), la fréquence cardiaque en position debout (*p* = 0,04), la variation (delta) de la fréquence cardiaque (*p* = 0,01) et les symptômes s’étaient détériorés (*p* = 0,02).

**Conclusions:**

Les vêtements de compression abdominale disponibles sur le marché permettent de reduiré la fréquence cardiaque et d’atténuer les symptômes immédiats et apres plusieurs heures d’utilisation. Chez les patients atteints de STOP, les vêtements de compression abdominale pourraient représenter une solution de rechange acceptable aux vêtements de compression montant jusqu’à la taille.

Postural orthostatic tachycardia syndrome (POTS) is a debilitating autonomic nervous system disorder that primarily impacts female patients.^1^ Patients with POTS experience orthostatic intolerance defined as a heart rate (HR) increase of ≥ 30 beats per minute (bpm) within 10 minutes of upright posture, in association with chronic orthostatic symptoms, and in the absence of orthostatic hypotension (decrease of > 20/10 mm Hg).^2–4^

There are no approved medications for use in POTS and patients are treated with a combination of off-label pharmacologic and nonpharmacologic approaches. Compression garments are a frequently prescribed treatment for POTS. Compression garments provide pressure to the abdomen and legs, shifting pooled blood back to the heart. We have previously demonstrated that compression of the abdomen and thigh using a proof-of-concept garment reduces HR and improves symptoms in an acute laboratory setting.^5^ We also demonstrated that commercially available waist-high compression garments are effective in a real-world community setting.^6^ Waist-high compression tights may not always be a practical option, especially during hot weather or when there are specific fashion requirements. Commercially available abdominal compression garments are an alternative, but they have not been studied in patients with POTS. Research has tested inflatable abdominal bands in POTS^7^ and related conditions of orthostatic intolerance,^8^ but these are not a practical option for everyday use.

In this study we tested commercially available abdominal compression garments as a treatment for POTS in a community setting. We hypothesized that abdominal compression garments would reduce HR and improve symptoms in patients with POTS.

## Methods

### Overall study design

This clinical trial received ethics approval from the Conjoint Health Research Ethics Board at the University of Calgary (ID: REB20–2224) and is a registered trial on ClinicalTrials.gov (ID: NCT04881318). This was a before-vs-after clinical trial conducted in a virtual setting with no in-person participation. Participants held medications that could augment HR and blood pressure on the study day. The study protocol duration was 1 day. Participants completed an electronic informed consent process through the **R**esearch **E**lectronic **D**ata **Cap**ture (REDCap) platform hosted at the University of Calgary.^9^ Participants were mailed a study package with a Holter monitor (Bittium Faros 180; Bittium Corp, Oulu, Finland) along with step-by-step study instructions and study worksheets. The Holter monitor recorded a 3-lead electrocardiogram as well as 3-dimensional accelerometer data. Prior to the study day, but after receiving the study materials, each participant attended a virtual meeting with a member of the research team to review the use of the Holter monitor and the steps to complete the study protocol. A member of the research team was always available for the participant to contact if they had any questions or concerns. The methods for this study have been detailed in a previous study.^6^

### Study participants

**Figure 1.**
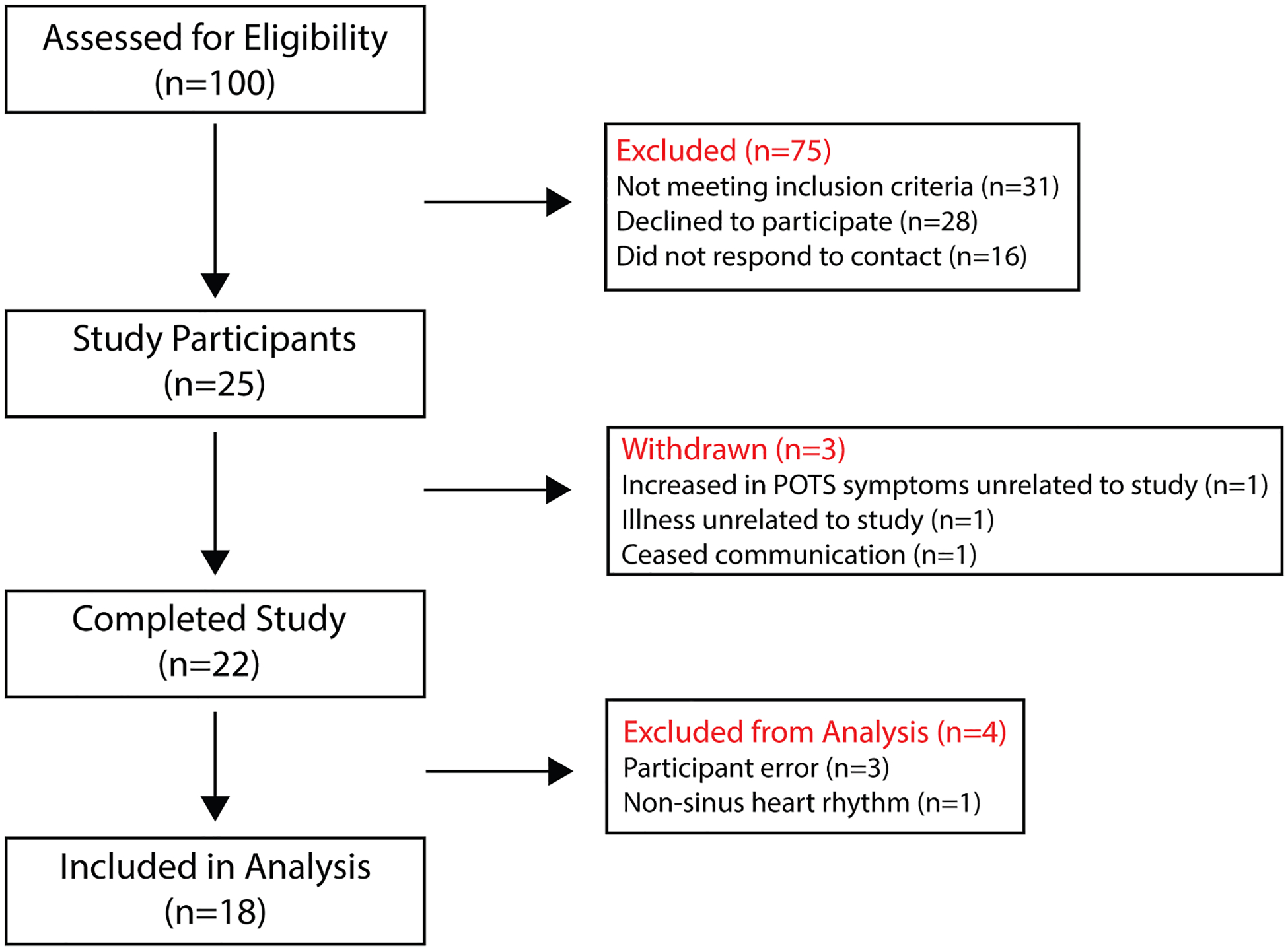
Study flow diagram. A total of 25 participants were enrolled in the study, 22 participants completed the study protocol, and 18 were included in the analysis. POTS, postural orthostatic tachycardia syndrome.

We attempted to contact 100 participants for participation in this study (Supplemental Table S1). Twenty-five participants between 19 and 54 years of age were enrolled in the study from May 2021 to May 2023. The participants were recruited from the Calgary Autonomic Investigation and Management Clinic and from the University of Calgary’s “Participate in Research” Web site. Participants had a physician’s diagnosis of POTS, defined as a ≥ 30-bpm increase in heart rate within 10 minutes of upright posture, in association with orthostatic symptoms and in the absence of orthostatic hypotension (> 20/10 mm Hg). Exclusion criteria for the study were: overt cause for postural tachycardia that precluded POTS diagnosis; somatization or severe anxiety; pregnancy (self-reported); inability to tolerate compression garments for the duration of the study; and not owning an abdominal compression garment. Three participants withdrew from the study before completing the protocol (1 due to a worsening of POTS symptoms unrelated to the study, 1 due to illness unrelated to the study, and 1 who completed the informed consent process but ceased correspondence with study investigators; Supplemental Table S2). Of the 22 participants who completed the study protocol, 4 were removed during data analysis (1 due to a non-sinus heart rhythm [junctional rhythm], and 3 due to participant error when completing the study protocol). The specific participant errors included: 1) participant removed the compression garment after orthostatic vitals #2 and completed orthostatic vitals #3 without compression instead of with compression; 2) participant only waited for 10 minutes after putting on the compression garment instead of 30 minutes before completing orthostatic vitals #2; and 3) participant failed to record orthostatic vitals #1 on the Holter monitor. Therefore, 18 participants were included in the analysis (Fig. 1).

### Types of compression garments

**Figure 2.**
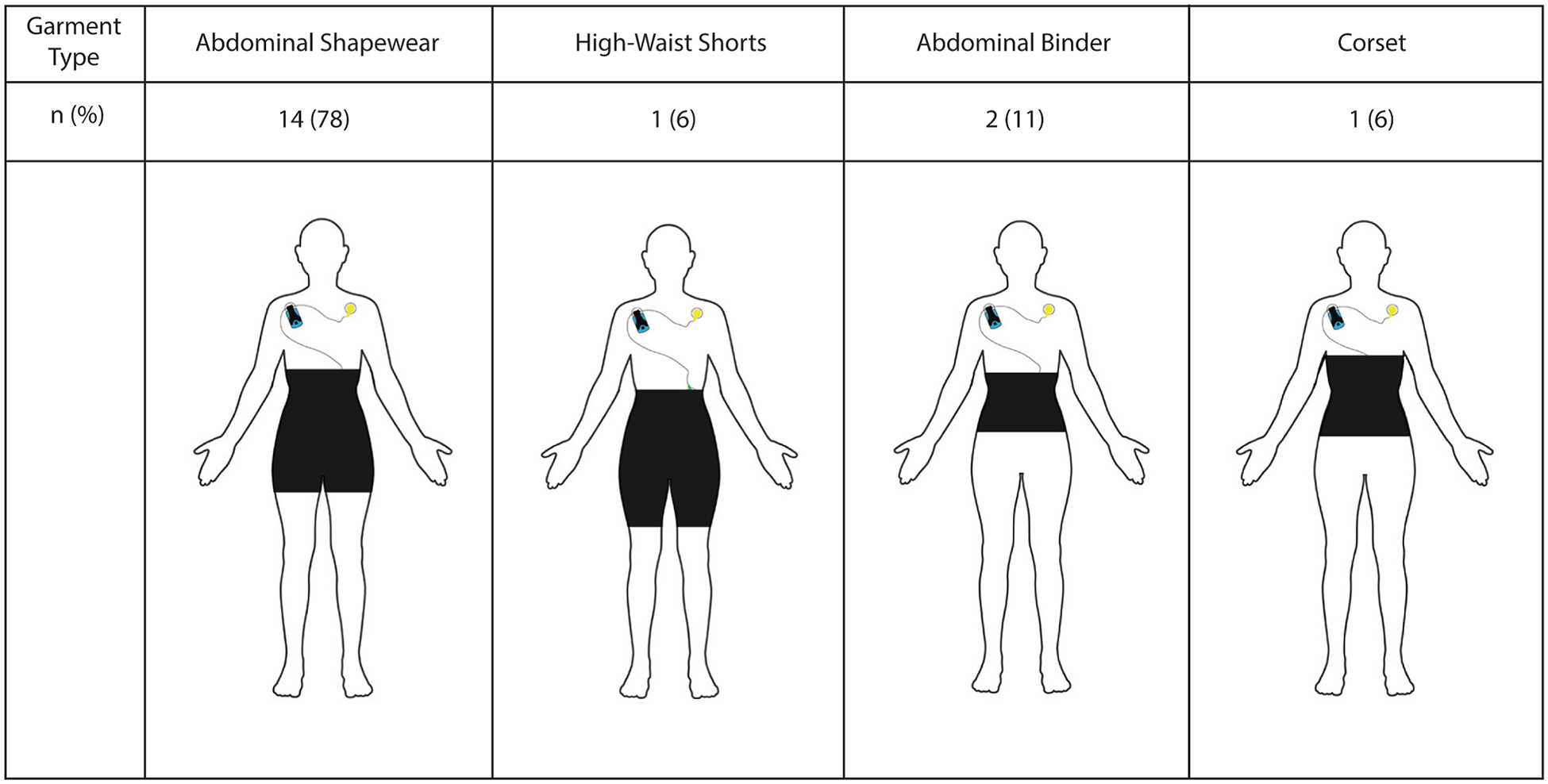
Compression garment diagram. Participants wore abdominal compression garments that included abdominal shapewear, compression shorts, abdominal binders, and corsets. For the duration of the study, each participant wore only one of these garments. Garment selection was based on the type of garment owned by the participant prior to the study.

Participants provided their own abdominal compression garment (shapewear, shorts, binder, or corset) for the study and information about the type of compression garment was recorded. Abdominal shapewear extends from the upper thigh to just below the rib cage, abdominal shorts from the waist to the thigh, abdominal binder on the abdomen without any thigh compression, and corset on the torso from the top of the hips to the bottom of the rib cage. A diagram of these garments in shown in Figure 2.

### Study day

Participants followed the step-by-step written instructions provided by the research team when completing the study day. Participants began by connecting the Holter monitor to 3 electrodes placed below the left and right midclavicular lines, as well as the left abdomen below the bottom rib. Participants then took medications not held (medications that did not augment HR and blood pressure). The participants continued other nonpharmacologic treatments (eg, increased salt and water). Thirty minutes after taking medications not held (or after putting on the monitor if participants were not taking any medications), each participant completed the first standing test. With the Holter monitor turned on, the participant took a supine position and a 3-minute baseline recording was obtained. The participant pressed the marker button on the Holter monitor at the beginning of the supine baseline to mark the start of the test. The participant also recorded this time on the study worksheet. Immediately after the 3-minute baseline, the participant stood for 10 minutes. The participant was then instructed to remain still during standing. The participant was also instructed to cease standing and immediately lie down if they felt like they were going to faint. After the 10-minute standing test, the participant performed a self-assessment of symptoms using the **V**anderbilt **O**rthostatic **S**ymptom **S**cale (VOSS; no symptoms defined as 1/10). After the study, symptom ratings of 1 were converted to 0 to align with conventional VOSS scoring (range 0–90).

**Figure 3.**
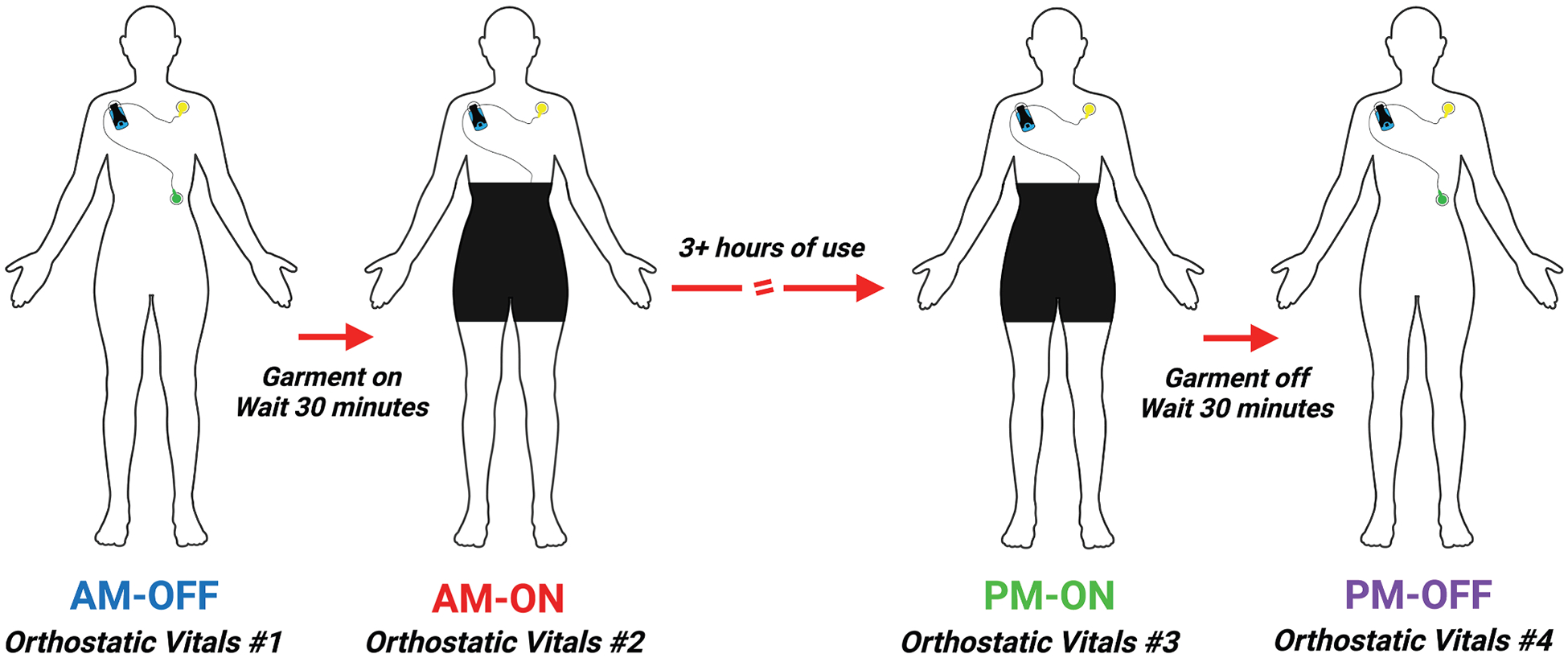
Study day diagram. Participants completed four 10-minute active stand tests on each study day. Two tests were competed with and without compression in the morning (AM-ON and AM-OFF) and the other 2 tests were completed in the afternoon or evening with and without compression (PM-ON and PM-OFF). AM-OFF, compression garment off in the morning; AM-ON, compression garment on in the morning; PM-OFF, compression garment off in the afternoon.

The study day is shown in Figure 3. After the first standing test (AM-OFF), the participant then put on their compression garment. Thirty minutes after putting on the compression garment, the participant completed the second standing test (AM-ON), using the same protocol previously described. A 30-minute period between putting on the garment and the second standing test was included to ensure that any HR elevation or symptom exacerbation from putting on the garment had resolved. After the AM-ON test, the participant then wore the compression garment for a minimum of 3 hours. The participant was instructed to continue with their daily activities while wearing the garment. Prior to removing the garment, and after a minimum of 3 hours, the participant completed the third standing test (PM-ON). After the PM-ON test, the participant then removed the compression garment. Thirty minutes after removing the garment, the participant completed the final standing test (PM-OFF). The PM-OFF standing test was included because HR is lower later during the daytime due to diurnal variability.^10^ After the PM-OFF test, the participant removed the Holter monitor and the study day was over. After the study, the participant was sent an online REDCap survey. The participant returned the materials to the research laboratory using a prepaid envelope.

### Signal analysis

**Figure 4.**
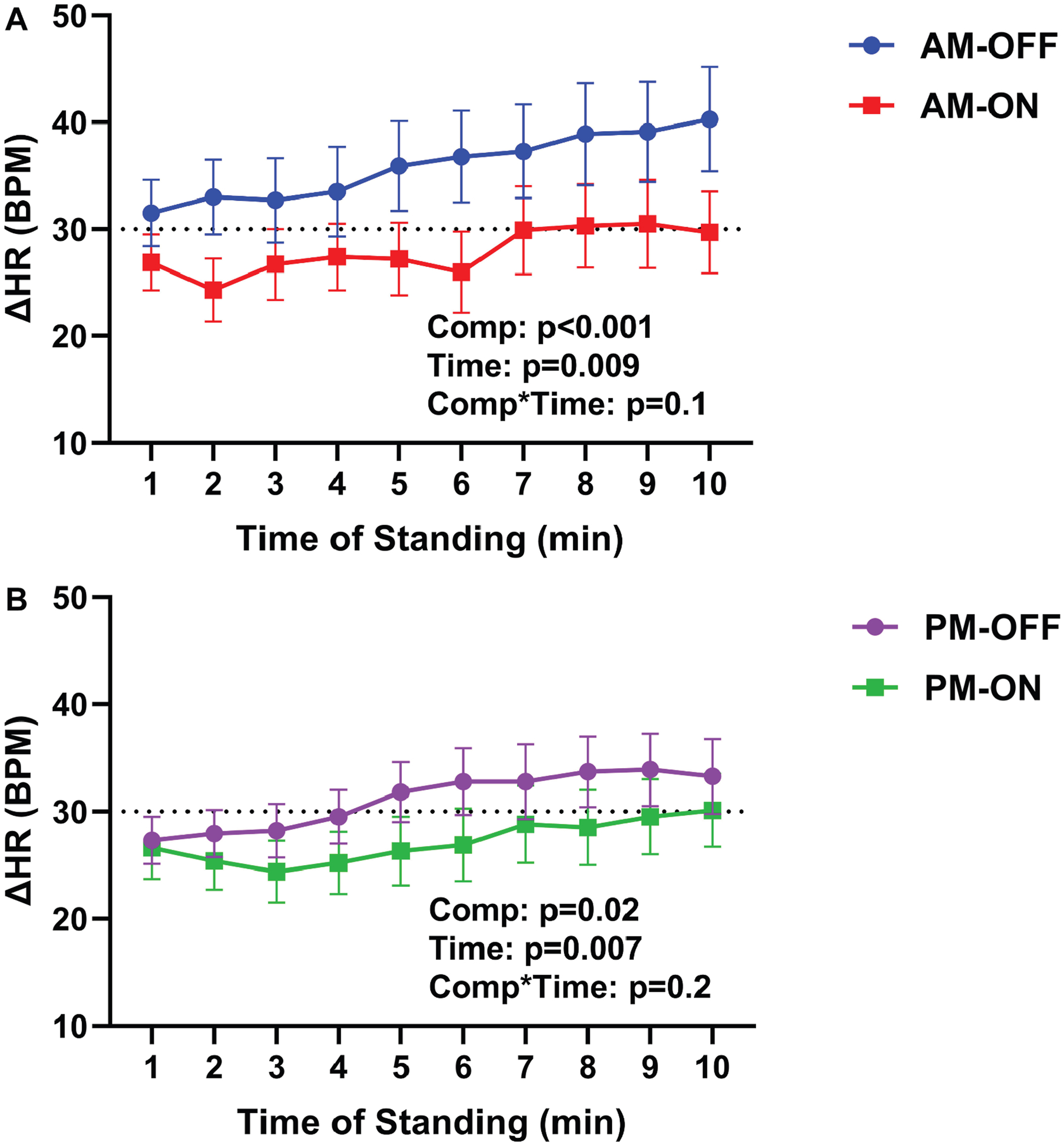
Heart rate. (**A**) ΔHR (standing – baseline) during the 10 minutes of standing of AM-ON compared with AM-OFF. (**B**) ΔHR (standing – baseline) during the 10 minutes of standing of PM-ON compared with PM-OFF. AM-OFF, compression garment off in the morning; AM-ON, compression garment on in the morning; HR, heart rate; PM-OFF, compression garment off in the afternoon.

All data files were recorded at a minimum of 125 Hz. Data files in European Data Format were downloaded from the Holter monitor and imported into a custom MATLAB (The MathWorks, Natick, MA) program for analysis. Data files were manually reviewed and signal artifacts were removed. The participant entered markers in the file and the time was written on the worksheet by the participant. The accelerometer signals were used to confirm the location of the standing tests within the recording. The RR intervals were obtained from the electrocardiographic recording and converted to HR. Analyzed data were stored in a REDCap database at the University of Calgary.^9^ Supine baseline HR was calculated by averaging the HR for the 3 minutes of baseline. HR during each minute of standing was calculated by averaging 60-second intervals of HR. Maximum HR during standing was taken from the minute with the highest average HR between minutes 6–10 of standing. For participants who stopped standing prior to the end of the 10-minute test, the minute with highest HR before the stand test ended was used. The last minute before the standing test ended was not always used because sometimes a vasovagal reaction developed leading to a decrease in HR. Delta (Δ) values were calculated by subtracting the supine baseline value from the minute with the highest HR during standing. For participants who stopped standing early (Supplemental Table S3), the mean HR value for each minute of standing that was missing was extrapolated from the last minute of standing. These data were used as shown in Figure 4. Orthostatic index was calculated from the area under the curve of mean RR interval vs time during standing, using the trapezoidal method for each minute while upright.^5,11^ The orthostatic index is larger when HR is lower (longer RR interval) and stand duration is longer, and smaller when HR is higher (shorter RR interval), and stand duration is shorter. A shorter length of stand will reduce the area under the curve because the time duration is shorter.

### Statistical analysis

Data were exported from REDCap^9^ and imported into SPSS version 28 (IBM Corp, Armonk, NY) for analysis. The primary analysis was HR during AM-OFF compared with AM-ON. Secondary analyses included HR during PM-OFF compared with PM-ON, as well as orthostatic index, percent change of HR, and symptoms during AM-OFF compared with AM-ON and PM-OFF compared with PM-ON. Diurnal HR variability was evaluated by comparing HR on AM-OFF compared with PM-OFF. The distribution of each variable was tested using Shapiro-Wilk normality tests. Paired *t* tests were used to compare AM-OFF and AM-ON, PM-OFF and PM-ON, AM-OFF and PM-OFF, and AM-ON and PM-ON for normally distributed variables, and Wilcoxon signed-rank tests were used for non—normally distributed variables. Two-factor repeated-measures analysis of variance testing was used to compare compression (on/off) and time during each minute of standing for AM-OFF vs AM-ON and PM-OFF vs PM-ON. Continuous data are presented as median (25th-75th percentile) and categorical data are presented as percent (n). *P* < 0.05 was considered statistically significant.

### Sample size calculation

Preliminary data with a proof-of-concept compression garment demonstrated a reduction in ΔHR with abdominal compression of 11 bpm when compared with no compression, with a standard deviation of 11 bpm.^5^ The HR reduction with commercial abdominal compression garments is not known. We anticipated that a clinically meaningful reduction in HR would be 10 bpm, with an anticipated standard deviation of 15 bpm. With the previously mentioned assumptions for a paired test of continuous data and a 0.05 two-sided significance level, a sample size of 20 POTS patients would allow for 80% power to detect this difference.

## Results

### Participants

Eighteen female patients with physician-diagnosed POTS completed the study day and were included in the analysis (Fig. 1). The mean age of participants was 37 ± 2 years with a body mass index of 24 ± 1 kg/m^2^. Most participants (n = 16, 89%) identified their race as White, 1 identified as Indigenous, and 1 identified multiple-race White. Most participants were taking at least 1 medication that modulated HR and/or blood pressure, including beta-blockers (56%, n = 10), ivabradine (11%, n = 2), midodrine (22%, n = 4), methyldopa (11%, n = 2), and pyridostigmine (11%, n = 2; Supplemental Table S4). These medications were held on the study day except for 1 participant, who did not hold methyldopa due to significant side effects when she had previously held this medication. This participant still met the POTS HR criterion on the study day (ΔHR 45 bpm on AM-OFF).

### Compression garment use before the study

Prior to the study, all participants (n = 18) reported regular use of compression garments to manage their POTS symptoms. Participants were asked which type of garment they wore most often: 56% (n = 10) reported abdominal compression (shapewear or shorts); 28% (n = 5) reported waist-high tights; and 16% (n = 3) reported knee-high or thigh-high stockings. Participants were also asked how often they used the garment: 39% (n = 7) reported only when doing specific activities; 28% (n = 5) reported every day; 17% (n = 3) reported every day for only part of the day; and 17% (n = 3) reported only when symptoms were worse.

### Compression garment use on study day

Most participants used abdominal shapewear (80%, Fig. 1). The garment was worn for a median duration of 5.5 (4.5–7.3) hours.

### Stand duration

Some stands were < 10 minutes due to presyncopal symptoms and participant discontinuation of the standing test. On AM-OFF, 3 stands were < 10 minutes (2, 3, and 8 minutes); on AM-ON, 2 stands were < 10 minutes (9 and 9 minutes); on PM-OFF, 1 stand was < 10 minutes (8 minutes); and, on PM-ON, 1 stand was < 10 minutes (8 minutes).

### AM-ON vs AM-OFF

**Figure 5.**
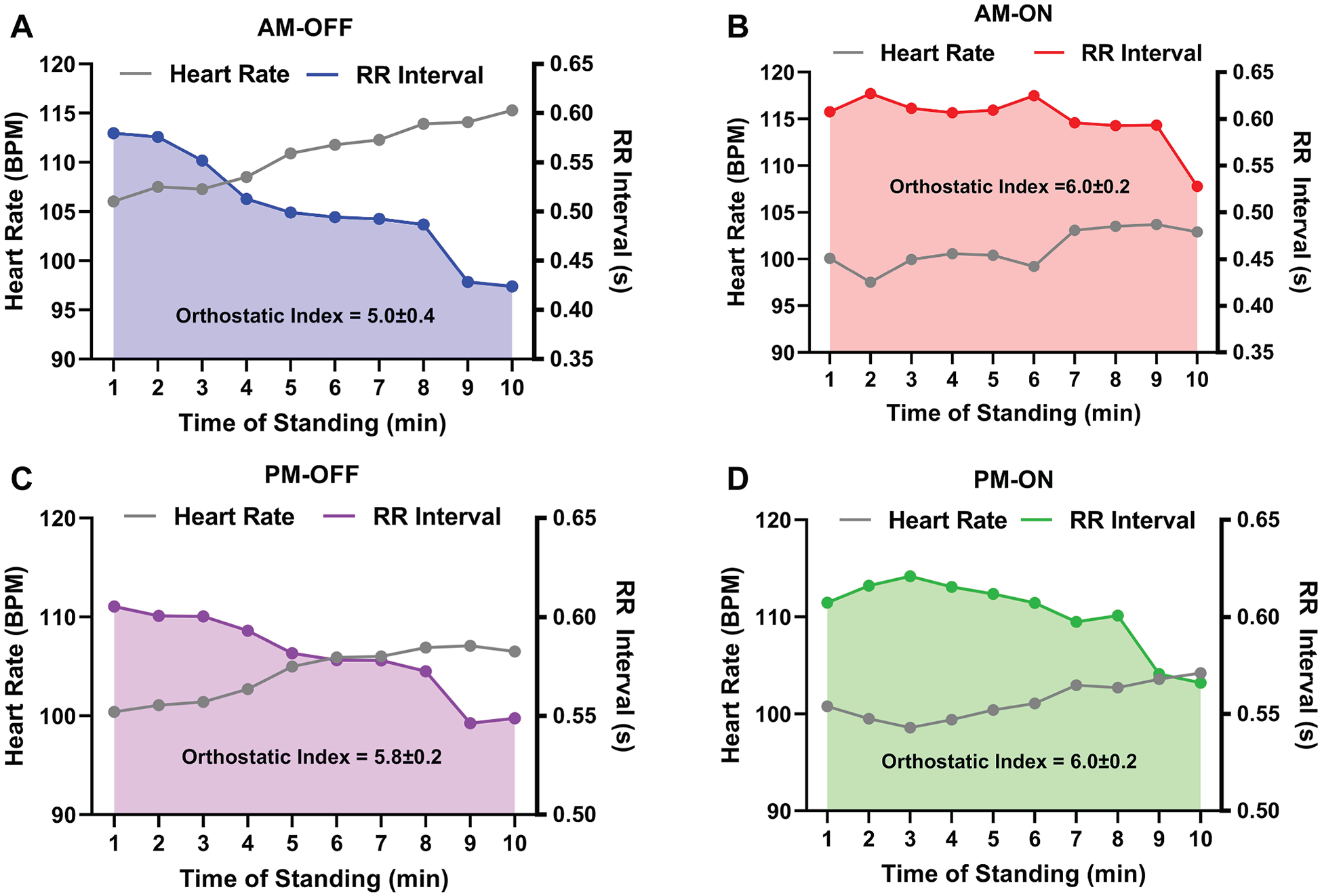
Orthostatic index. Orthostatic index (left y axis) and RR interval (right y axis) during AM-OFF (**A**), AM-ON (**B**), PM-OFF (**C**), and PM-ON (**D**). AM-OFF, compression garment off in the morning; AM-ON, compression garment on in the morning; PM-OFF, compression garment off in the afternoon; PM-ON, compression garment on in the afternoon.

Supine baseline HR was not different between AM-ON and AM-OFF (*P* = 0.5; Table 1). Stand HR (103 [91–114] vs 118 [99–134], *P* = 0.002) and ΔHR (27 [19–42] vs 41 [26–48], *P* < 0.001) were reduced during AM-ON compared with AM-OFF (Fig. 4A). Percent change in HR from supine to standing was smaller during AM-ON compared with AM-OFF (*P* < 0.001), and orthostatic index was larger during AM-ON compared with AM-OFF (*P* = 0.02; Fig. 5). VOSS symptoms were also reduced during AM-ON compared with AM-OFF (29 [16–45] vs 35 [25–60], *P* = 0.005; Supplemental Table S5).

### PM-ON vs PM-OFF

Supine baseline HR was not different between PM-ON and PM-OFF (74 [63–86] vs 73 [65–84], *P* = 0.4; Table 1). Stand HR (106 [91–122] vs 110 [98–121], *P* = 0.04) and ΔHR (29 [24–38] vs 35 [26–45], *P* = 0.01) were reduced during PM-ON compared with PM-OFF (Fig. 4B). Percent change in HR from supine to standing was smaller during PM-ON compared with PM-OFF (*P* = 0.02), and orthostatic index was larger during PM-ON compared with PM-OFF (*P* = 0.04). VOSS symptoms were also reduced during PM-ON compared with PM-OFF (26 [14–41] vs 32 [17–48], *P* = 0.02).

### Compression effects over several hours

Supine baseline HR (*P* = 0.7), stand HR (*P* = 0.9), ΔHR (*P* = 0.8), percent change in HR (*P* = 0.8), and orthostatic index (*P* = 0.9) were similar during AM-ON and PM-ON (Supplemental Table S6). VOSS symptoms were also similar between AM-ON and PM-ON (*P* = 0.6).

### Diurnal HR variability

PM-OFF stand HR (110 [98–1211] vs 118 [99–134], *P* = 0.07) and ΔHR (35 [26–45] vs 41 [26–48], *P* = 0.1) trended lower than AM-OFF, but these values were not significantly different (Supplemental Table S6).

## Discussion

This trial has demonstrated that commercially available abdominal compression garments are effective at reducing HR below the POTS criterion of a ≥ 30-bpm increase and improving symptoms in patients with POTS. The trial took place in a community setting with patients wearing their own garments and participating in their daily routines, simulating everyday life.

### Effects of abdominal compression

At rest, the splanchnic circulation receives 25%-30% of the cardiac output.^12^ Upon standing, approximately 500 mL of fluid shifts from the thorax into the abdomen, with some fluid moving to the legs.^13^ Some patients with POTS have further increased blood pooling in the splanchnic region both when upright and at rest, compared with healthy controls.^14,15^ Therefore, compression applied to the abdomen targets the majority of this blood pooling and provides effective reduction in HR and symptoms, even without compression of the full lower extremity. Research has demonstrated a reduction in the inferior vena cava diameter with the application of inflatable abdominal compression in patients with orthostatic intolerance, demonstrating a change in the capacitance of the splanchnic circulation with the addition of compression.^16^ In our study, the abdominal compression garment reduced the orthostatic HR increase upon standing by 14 bpm, and significantly reduced symptoms. In our initial in-laboratory trial, abdominal compression reduced the orthostatic HR increase by a mean of 11 bpm.^5^ These data suggest that abdominal compression works as well in the “real world” as it does in a laboratory setting.

A previous study of orthostatic intolerance showed that, when an inflatable abdominal band was applied to patients undergoing head-up tilt, blood pressure increased and orthostatic symptoms improved.^8^ An earlier study investigated splanchnic venous compression with an inflatable abdominal band, in combination with propranolol, in patients with POTS.^7^ That study showed a different result than we did in the current study. The compression helped to prevent the blood pressure decrease from propranolol, but no HR or blood pressure effects were observed. The form factor of their inflatable binder is different from that of the compression garments, with the latter likely providing more pelvic compression. This is a possible explanation for the differences in HR response. In our earlier in-laboratory trial, we demonstrated that participants with lower initial orthostatic HR increases had fewer benefits from compression.^5^ In our recent trial of full-length compression tights, however, additional HR benefit was seen when adding compression to medications.^6^ Future research could investigate commercially available abdominal compression garments in combination with medications to determine whether there is an increased HR and symptom benefit with the addition of compression.

### Benefits over several hours

When the compression garment was removed after several hours of use, both HR and symptoms increased. This occurred despite the diurnal reduction in HR that occurred later in the day and demonstrates that the garment was delivering sustained benefits throughout the period of use. This shows that abdominal compression can be used throughout the day for HR and symptom improvement.

### Abdominal compared with full lower body compression

In our study, the orthostatic HR increase was reduced by 14 bpm, compared with our recent study demonstrating a reduction of 20 bpm with full waist-high compression tights.^6^ This is consistent with findings from our in-laboratory trial, demonstrating increased benefit with full waist-high compression compared with abdominal only.^5^ Although not as beneficial as the full waist-high compression, abdominal compression still led to significant improvement compared with no compression. Research in orthostatic hypotension has also demonstrated similar findings, with full waist-high compression providing the most benefit in blood pressure preservation, followed by abdominal compression.^17^ Despite the slightly reduced benefit of abdominal-only compression, the strong advantage is the increased tolerance in hot weather, including during the summer and in warmer climates. The abdominal-only garment provides compression benefit while reducing the risk of overheating. In a separate qualitative interview study conducted by our group, we found that environmental conditions, including heat, were an important factor in the decision to use compression or not.^18^ In warmer weather patients, may be more likely to use abdominal-only compression and achieve some benefits. In addition, the visible nature and colour of waist-high compression tights may not be a suitable option in specific situations (eg, when wearing a dress or shorts). The potential advantages of abdominal-only compression over full tights are also reflected in the participant survey results where most participants chose to wear abdominal-only compression most often.

### Clinical implications

Patients with POTS may find limitations with conventional waist-high compression tights. Particularly in the summer and in warm climates these garments can cause overheating and discomfort. Abdominal compression garments may be a good alternative in these cases and may help to facilitate use of some compression.

### Limitations

Participants used their own compression garments for this study, and not a standardized garment. There is variability in the strength and style of abdominal compression garments. Most commercially available abdominal compression garments do not provide a pressure rating, and we therefore were unable to describe the pressure ratings of the garments.

Each participant acted as their own control, and this allowed us to assess the benefit of abdominal compression using different types of garments. In addition, it was not possible to blind the participants to the compression garment intervention in the study. All participants were diagnosed with POTS using the diagnostic criteria of orthostatic tachycardia (≥ 30 bpm within 10 minutes of upright posture) in the absence of orthostatic hypotension (decrease of > 20/10 mm Hg). During the study, however, blood pressure was not specifically measured, and it is possible that a participant could have experienced an isolated episode of orthostatic hypotension.

## Conclusions

Compression of the abdomen applied using commercially available abdominal compression garments reduced HR and improved symptoms in patients with POTS. These benefits were seen both acutely, and after several hours of use. Abdominal compression garments should be offered as a compression garment alternative for the treatment of POTS.

## Supplementary Material

1

To access the supplementary material accompanying this article, visit the online version of the *Canadian Journal of Cardiology* at www.onlinecjc.ca and at https://doi.org/10.1016/j.cjca.2025.11.038

## Figures and Tables

**Table 1.** Heart rate, orthostatic index, and symptoms

Measure	AM-OFF	AM-ON	AM-OFF vs AM-ON, *P* value	PM-OFF	PM-ON	PM-OFF vs PM-ON, *P* value
Baseline HR (bpm)	74 (63–88)	74 (64–81)	0.5	73 (65–84)	74 (63–86)	0.4
Stand HR (bpm)	118 (99–134)	103 (91–114)	0.002*	110 (98–121)	106 (91–122)	0.04*
ΔHR (bpm)	41 (26–48)	27 (19–42)	< 0.001*	35 (26–45)	29 (24–38)	0.01*
Percent ΔHR (%)	49 (37–84)	35 (26–38)	< 0.001*	51 (30–68)	43 (32–48)	0.02*
Orthostatic index (min/s)	5.1 (4.4–6.0)	6.0 (5.5–6.60)	0.02*	5.8 (5.3–6.5)	5.9 (5.2–6.9)	0.04*
VOSS (arbitrary units)	35 (25–60)	29 (16–45)	0.005*	32 (17–48)	26 (14–41)	0.02*

Data expressed as median (25th-75th percentile). Paired *t* tests were used to compare normally distributed variables, and Wilcoxon. signed-rank tests were used to compare non—normally distributed variables.

AM-OFF, compression garment off in the morning; AM-ON, compression garment on in the morning; HR, heart rate; PM-OFF, compression garment off in the afternoon; PM-ON, compression garment on in the afternoon; VOSS, Vanderbilt Orthostatic Symptom Score.

* Statistically significant (*P* < 0.05).
