# Supplementary material for: Abdominal-only Compression Garments Reduce Orthostatic Tachycardia and Improve Symptoms in Patients With Postural Orthostatic Tachycardia Syndrome
Source: Can J Cardiol. Author manuscript; Available in PMC 2026 Jul 15. (PMC13370740; doi:10.1016/j.cjca.2025.11.038)
Supplement: 1 [file NIHMS2193694-supplement-1.pdf]

Supplemental Table S1. Patients contacted for participation in the abdominal compression arm of the study.

Disposition	Number of patients (n)
Did not respond to researcher contact	16
Declined to participate	28
Did not meet the inclusion criteria	31
Enrolled in the abdominal compression arm	25
Total patient recruitment attempts	100

Supplemental Table S2. Adverse Events Leading to Withdrawal From Study

**Adverse Event**	**Number of participants (n)**
Ceased communication with study team after completing informed consent process	1
Illness exacerbation unrelated to study	1
POTS symptom exacerbation unrelated to study	1

Supplemental Table S3. Participants who stopped standing before 10 minutes during each of the stand tests. Participants were instructed to cease standing if they experienced significant pre-syncopal symptoms or anticipated syncope.

Standing Test	Participants who stopped standing early and timing
AM-OFF	n=2 participants at minute 3
	n=1 participant at minute 9
AM-ON	n=2 participants at minute 10
PM-OFF	n=1 participant at minute 8
PM-ON	n=1 participant at minute 8

Supplemental Table S4. Participant medications and holding during the study day.

Participant	Beta-blocker		Midodrine		Fludrocortisone		Ivabradine		Pyridostigmine		Methyldopa		Stimulant		SSRI/SNRI	
	**P**	**H**	**P**	**H**	**P**	**H**	**P**	**H**	**P**	**H**	**P**	**H**	**P**	**H**	**P**	**H**
1	Yes	Yes	Yes	Yes	No		No		No		Yes	Yes	No		No	
2	Yes	Yes	Yes	Yes	No		No		No		No		No		No	
3	No		No		No		No		No		No		No		No	
4	Yes	Yes	No		No		No		No		No		No		No	
5	Yes	Yes	No		No		No		No		No		No		No	
6	No		Yes	Yes	No		No		Yes	Yes	No		No		No	
7	Yes	Yes	No		No		No		No		No		No		No	
8	No		No		No		No		No		No		No		No	
9	Yes	Yes	No		No		No		No		No		No		No	
10	Yes	Yes	No		No		No		Yes	Yes	No		No		Yes	No
11	No		No		No		No		No		No		No		No	
12	Yes	Yes	No		No		No		No		No		No		No	
13	No		No		No		Yes	Yes	No		No		Yes	No	No	
14	No		No		No		No		No		No		No		Yes	No
15	No		No		No		Yes	Yes	No		No		No		No	
16	Yes	Yes	No		No		No		No		No		No		No	
17	No		Yes	Yes	No		No		No		Yes	No*	No		No	
18	Yes	Yes	No		Yes	No	No		No		No		No		No	

P: Prescribed to the participant. H: Patient held this medication on the study day.

*Participant was unable to hold medication due to side effects

Supplemental Table S5. Changes in Individual Vanderbilt Orthostatic Symptom Score (VOSS) Symptoms.

**VOSS Symptom**	**AM-OFF**	**AM-ON**	**P-value AM-OFF vs AM-ON**		**PM-OFF**	**PM-ON**	**P-value PM-OFF vs PM-ON**	
			**Unadjusted**	**Adjusted***			**Unadjusted**	**Adjusted***
Mental Clouding	5.5 (1.5, 7.3)	4.5 (1.5, 6.0)	**0.04**	0.4	3.5 (3.0, 7.0)	3.5 (2.0, 5.3)	**0.007**	0.06
Blurry Vision	3.0 (1.5, 6.0)	2.0 (0.0, 3.3)	**0.008**	0.07	2.5 (0.0, 4.3)	2.0 (0.0, 3.0)	**0.02**	0.2
Shortness of Breath	4.5 (0.0, 7.0)	3.0 (0.0, 5.0)	**0.04**	0.4	3.0 (1.5, 6.3)	3.0 (0.0, 5.0)	0.1	0.9
Rapid Heartbeat	5.0 (2.8, 7.0)	3.5 (1.5, 5.3)	**0.007**	0.06	3.5 (1.5, 6.0)	3.0 (0.0, 6.3)	0.3	1.0
Tremulousness	5.0 (2.0, 6.3)	3.5 (0.0, 5.0)	**0.005**	**0.045**	4.5 (2.0, 6.3)	4.0 (0.0, 5.3)	**0.03**	0.3
Chest pain	4.5 (2.0, 6.3)	3.5 (1.5, 5.0)	**0.02**	0.2	3.0 (1.5, 5.3)	3.0 (1.5, 4.0)	0.06	0.5
Headache	3.5 (2.0, 5.0)	3.0 (1.5, 4.5)	0.3	1.0	3.5 (1.5, 5.3)	2.5 (2.0, 4.3)	0.2	1.0
Lightheadedness	7.0 (3.0, 8.0)	4.5 (2.0, 7.0)	0.1	0.9	5.5 (3.0, 8.0)	3.5 (2.0, 5.5)	**0.004**	**0.04**
Nausea	4.0 (1.5, 6.5)	2.0 (0.0. 4.3)	**0.02**	0.2	2.0 (0.0, 5.3)	2.0 (0.0, 4.3)	0.09	0.8

*Adjusted p-value for 9 comparisons. Data are presented as median (25th, 75th percentile). Paired t-tests were used to compare normally distributed variables and Wilcoxon Signed-Rank tests were used to compare non-normally distributed variables.

Supplemental Table S6. AM-OFF compared to PM-OFF and AM-ON compared to PM-ON.

Measure	AM-OFF	PM-OFF	p-value	AM-ON	PM-ON	p-value
Baseline HR (bpm)	74 (63, 88)	73 (65, 84)	0.9	74 (64, 81)	74 (63, 86)	0.7
Stand HR (bpm)	118 (99, 134)	110 (98, 121)	0.07	103 (91, 114)	106 (91, 122)	0.9
Delta HR (bpm)	41 (26, 48)	35 (26, 45)	0.1	27 (19, 42)	29 (24, 38)	0.8
Percentage Δ HR (%)	49 (37, 84)	51 (30, 68)	0.08	35 (26, 38)	43 (32, 48)	0.8
Orthostatic Index (min/s)	5.1 (4.4, 6.0)	5.8 (5.3, 6.5)	0.09	6.0 (5.5, 6.60	5.9 (5.2, 6.9)	0.9
VOSS (arbitrary units)	35 (25, 60)	32 (17, 48)	0.2	29 (16, 45)	26 (14, 41)	0.6

Data are presented as median (25th, 75th percentile). Paired t-tests were used to compare normally distributed variables and Wilcoxon Signed-Rank tests were used to compare non-normally distributed variables.

Reporting checklist for randomised trial.

Based on the CONSORT guidelines.

Instructions to authors

Complete this checklist by entering the page numbers from your manuscript where readers will find each of the items listed below.

Your article may not currently address all the items on the checklist. Please modify your text to include the missing information. If you are certain that an item does not apply, please write "n/a" and provide a short explanation.

Upload your completed checklist as an extra file when you submit to a journal.

In your methods section, say that you used the CONSORTreporting guidelines, and cite them as:

Schulz KF, Altman DG, Moher D, for the CONSORT Group. CONSORT 2010 Statement: updated guidelines for reporting parallel group randomised trials

		Reporting Item	Page Number
**Title and Abstract**			
Title	#1a	Identification as a randomized trial in the title.	N/A
Abstract	#1b	Structured summary of trial design, methods, results, and conclusions	2
**Introduction**			
Background and objectives	#2a	Scientific background and explanation of rationale	4
Background and objectives	#2b	Specific objectives or hypothesis	4
**Methods**			
Trial design	#3a	Description of trial design (such as parallel, factorial) including allocation ratio.	5

Trial design	#3b	Important changes to methods after trial commencement (such as eligibility criteria), with reasons	N/A
Participants	#4a	Eligibility criteria for participants	5
Participants	#4b	Settings and locations where the data were collected	5
Interventions	#5	The experimental and control interventions for each group with sufficient details to allow replication, including how and when they were actually administered	6-7
Outcomes	#6a	Completely defined prespecified primary and secondary outcome measures, including how and when they were assessed	9
Outcomes	#6b	Any changes to trial outcomes after the trial commenced, with reasons	N/A
Sample size	#7a	How sample size was determined.	9
Sample size	#7b	When applicable, explanation of any interim analyses and stopping guidelines	N/A
Randomization – Sequence generation	#8a	Method used to generate the random allocation sequence.	
N/A			
Randomization – Sequence generation	#8b	Type of randomization; details of any restriction (such as blocking and block size)	
N/A			
Randomization - Allocation concealment mechanism	#9	Mechanism used to implement the random allocation sequence (such as sequentially numbered containers), describing any steps taken to conceal the sequence until interventions were assigned	N/A

Randomization - Implementation	#10	Who generated the allocation sequence, who enrolled participants, and who assigned participants to interventions	N/A
Blinding	#11a	If done, who was blinded after assignment to interventions (for example, participants, care providers, those assessing outcomes) and how.	N/A
Blinding	#11b	If relevant, description of the similarity of interventions	N/A
Statistical methods	#12a	Statistical methods used to compare groups for primary and secondary outcomes	9
Statistical methods	#12b	Methods for additional analyses, such as subgroup analyses and adjusted analyses	9
**Results**			
Participant flow diagram (strongly recommended)	#13a	For each group, the numbers of participants who were randomly assigned, received intended treatment, and were analysed for the primary outcome	10
Participant flow	#13b	For each group, losses and exclusions after randomization, together with reason	5-6
Recruitment	#14a	Dates defining the periods of recruitment and follow-up	5
Recruitment	#14b	Why the trial ended or was stopped	N/A
Baseline data	#15	A table showing baseline demographic and clinical characteristics for each group	N/A
Numbers analysed	#16	For each group, number of participants (denominator) included in each analysis and whether the analysis was by original assigned groups	N/A
Outcomes and estimation	#17a	For each primary and secondary outcome, results for each group, and the estimated effect	11-12

		size and its precision (such as 95% confidence interval)	
Outcomes and estimation	#17b	For binary outcomes, presentation of both absolute and relative effect sizes is recommended	N/A
Ancillary analyses	#18	Results of any other analyses performed, including subgroup analyses and adjusted analyses, distinguishing pre-specified from exploratory	11-12
Harms	#19	All important harms or unintended effects in each group (For specific guidance see CONSORT for harms)	N/A
**Discussion**			
Limitations	#20	Trial limitations, addressing sources of potential bias, imprecision, and, if relevant, multiplicity of analyses	15
Generalisability	#21	Generalisability (external validity, applicability) of the trial findings	15
Interpretation	#22	Interpretation consistent with results, balancing benefits and harms, and considering other relevant evidence	13-15
Registration	#23	Registration number and name of trial registry	5
**Other information**			
Interpretation	#22	Interpretation consistent with results, balancing benefits and harms, and considering other relevant evidence	13-15
Registration	#23	Registration number and name of trial registry	5
Protocol	#24	Where the full trial protocol can be accessed, if available	N/A
Funding	#25	Sources of funding and other support (such as supply of drugs), role of funders	17

None The CONSORT checklist is distributed under the terms of the Creative Commons Attribution License CC-BY. This checklist can be completed online using https://www.goodreports.org/, a tool made by the EQUATOR Network in collaboration with Penelope.ai
